# Cytology and Differential Diagnosis of Canine Spindle Cell Lipoma

**DOI:** 10.1002/vms3.70587

**Published:** 2025-10-15

**Authors:** Marta Santos, Ricardo Marcos, João Oliveira, Giancarlo Avallone

**Affiliations:** ^1^ Cytology and Haematology Diagnostic Services, Laboratory of Histology and Embryology, ICBAS—School of Medicine and Biomedical Sciences, University of Porto Porto Portugal; ^2^ Escola Universitária Vasco da Gama Coimbra Portugal; ^3^ Department of Veterinary Medical Sciences (DIMEVET) University of Bologna Bologna Italy

**Keywords:** canine, cytology, lipoma, spindle cell lipoma

## Abstract

Benign lipomatous tumours in dogs included the very common lipoma and its rare variants, such as spindle cell lipoma. Canine spindle cell lipomas have been seldom described, and their histopathological clues included the concomitant presence of mature adipose tissue and a population of spindle cells immersed in a fibromyxoid matrix. The cytological features of this lipocytic tumour have never been described in veterinary medicine. Herein, a cytological description of a spindle cell lipoma in a 3‐year‐old dog, for which 2 years follow‐up data were available is presented. Cytological smears were characterized by a mixture of mature adipocytes and pleomorphic spindle cells, including multinucleated cells in association with blood vessels, thick collagen fibres and small amount of mucinous matrix. On histopathology, the tumour exhibited some papillary growth and was composed mostly by mature adipose tissue enriched in blood vessels and a smaller number of spindle cells immersed in a matrix with ropey collagen and myxoid substance. Spindle cells were positive to vimentin and negative for muscle, neural and endothelial markers. The cytological findings in the present case paralleled descriptions for human spindle cell and pleomorphic lipomas, which are related variants. The abundance of the mature adipose tissue component and unremarkable follow‐up with no recurrence or metastases documented were compatible with a benign tumour. This case highlighted that a prompt diagnosis of sarcoma should be avoided when a cytological sample from a subcutaneous mass in a dog is characterized by a mixture of mature adipocytes and spindle cells in association with collagen bundles.

## Graphical abstract

A slowly growing large mass in the gluteal area was present in the gluteal area. Fine needle aspiration was performed, and special stains (oil red) were used. First cytological description of a spindle cell lipoma (confirmed by histopathology).

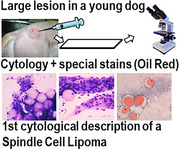

## 1 Introduction

In domestic animals, the benign tumours of adipose tissue include the frequent lipoma, infiltrative lipoma and variants characterized by the presence of additional components, such as fibrolipoma, angiolipoma, chondrolipoma and osteolipoma (Hendrick et al. 1998; Ramírez et al. 2010). These variants are rare tumours in the veterinary field (Hendrick 2017). Another benign and rare lipocytic tumour variant, named spindle cell lipoma, was also described in a case series of six dogs (Avallone et al. 2017). To the best of our knowledge no reference to this entity exists in veterinary cytology literature. Herein, we described, for the first time, the cytological features of a histological confirmed canine spindle cell lipoma, for which 2 years of follow‐up was available.

## 2 Case Presentation

**FIGURE 1:**
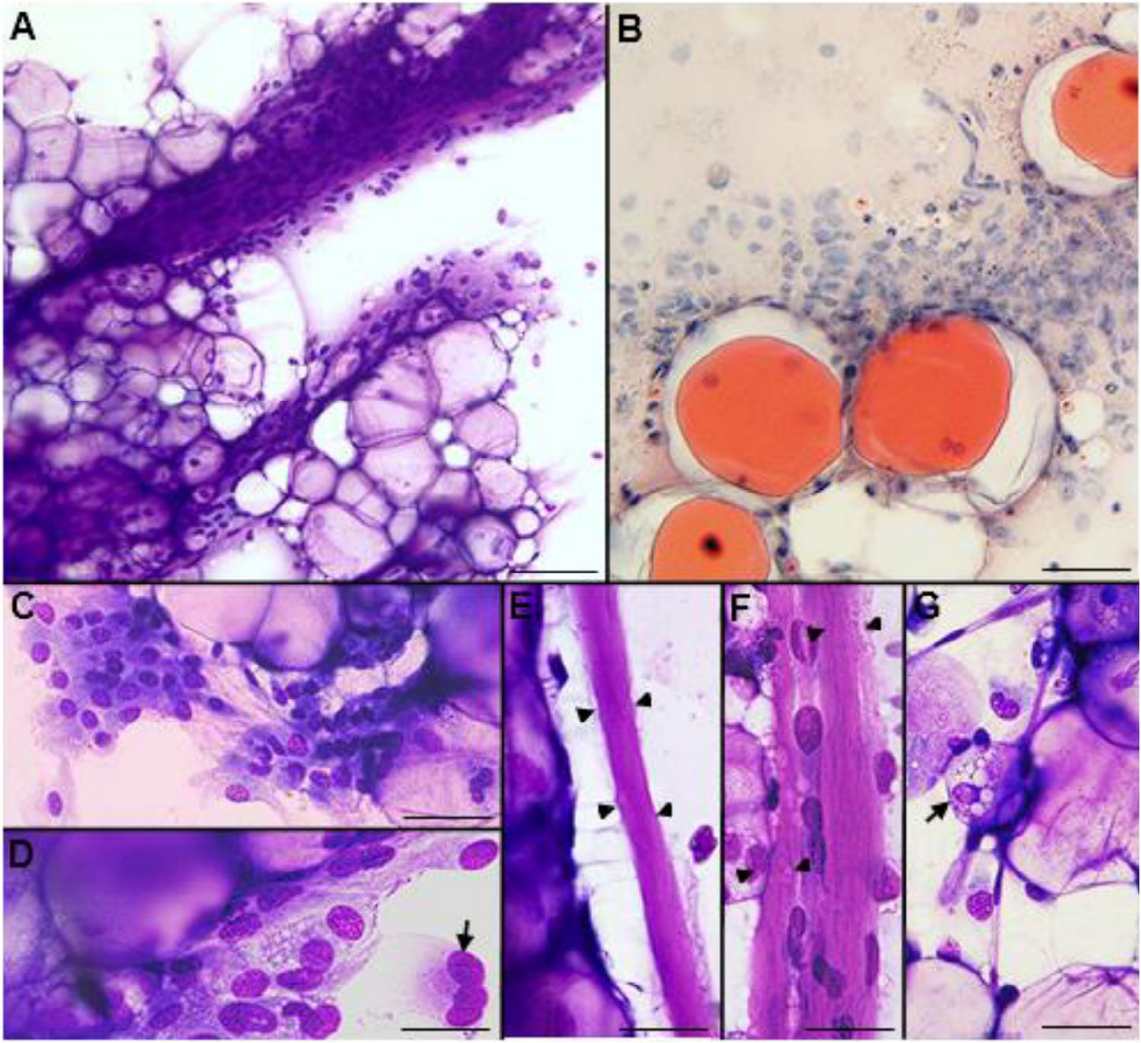
Cytological features of a canine spindle cell lipoma. (A) Hemacolor, aggregates of mature adipose tissue intermingled with a spindle cell population present in a fibromyxoid vascular matrix, bar = 70 µm. (B) Oil red O, fat vacuoles visualized with oil red O staining, bar = 40 µm. (C) Hemacolor, a mixture of mature adipocytes and mildly cohesive, bland spindle to stellate cells, bar = 50 µm. (D) Hemacolor, spindle cells with small cytoplasmic vacuoles and enlarged nuclei; a multinucleate cell with the nuclei arranged in a semicircle is present (arrow), bar = 25 µm. (E and F) Hemacolor, hyaline and eosinophilic collagen fibres (arrowheads) in close association with adipocytes and spindle cells, bar = 30 µm. (G) Hemacolor, mature adipocytes with delicate capillaries and a multivacuolate lipoblast‐like cell (arrow), bar = 30 µm.

A 3‐year‐old male Labrador retriever was presented to a private veterinary clinic with a 2‐month history of a progressively enlarging subcutaneous mass in the gluteal area. The physical examination, CBC and biochemical analysis were unremarkable. The clinician referred a non‐painful subcutaneous mass, with 6 cm in the largest diameter that presented well defined borders and soft texture. The referring clinician performed an ultrasound (US) examination of the gluteal mass and fine‐needle aspirations (FNA). The mass presented a heterogeneous pattern in US, and the cytological smears contained grossly glistening droplets and blood. Air‐dried smears were stained with Hemacolor (Merck, Darmstadt, Germany) and oil red O (OR) (Figure 1). The specimens were moderately cellular with slight blood contamination. A mixed population predominantly composed of mature adipocytes and spindle to stellate cells was present (Figure 1A). Large three‐dimensional groups of univacuolated adipocytes containing blood vessels were observed (Figure 1A). Cytoplasmic vacuoles were positive to OR (Figure 1B). Spindle cells arranged in clusters close to mature adipocytes were seen (Figure 1C,D). These cells were frequently associated with thick collagen fibres or capillaries and more rarely appeared isolated (Figure 1E,F). Spindle cells were mildly cohesive, with a light blue cytoplasm containing occasional small vacuoles with distinct margins and, more rarely, pink cytoplasmic globules (Figure 1C,D). Nuclei were pleomorphic, varying from pointed to round or oval or, more rarely, irregular, presenting 10–16 µm of diameter, with a reticular chromatin pattern. Inconspicuous nucleoli or a single small nucleolus and very rarely with nuclear pseudoinclusions were also observed. Binucleated and multinucleated cells (with nuclei arranged in a semicircle and mild nuclear atypia) also appeared (Figure 1D). In some cellular groups a myxoid matrix was intermingled with the spindle cells. No mitotic figures or necrosis were observed. Very few multivacuolated lipoblast‐like cells were rarely admixed with mature adipocytes (Figure 1G). Numerous foamy macrophages with ingested lipid (lipophages) and/or engulfing erythrocytes (hemosiderophages) were also observed. These findings were consistent with a lipomatous tumour, such as spindle cell lipoma, even though a low‐grade soft tissue sarcoma could not be completely excluded. A surgical excision with wide margins was recommended.

The gluteal mass was surgically removed. The lateral margins were well defined, but firm adhesion to the underlying skeletal muscles and extension to the deep surgical margins were evident. During trimming, a pale tan colour mass measuring 20 × 15 × 8 cm^3^ and characterized by solid areas, multilobulated frond‐like areas and papillary formations was observed. Samples of different areas (solid and papillary) were routinely processed, sectioned (5 µm) and stained with haematoxylin–eosin, Masson trichrome and Alcian blue.

**FIGURE 2:**
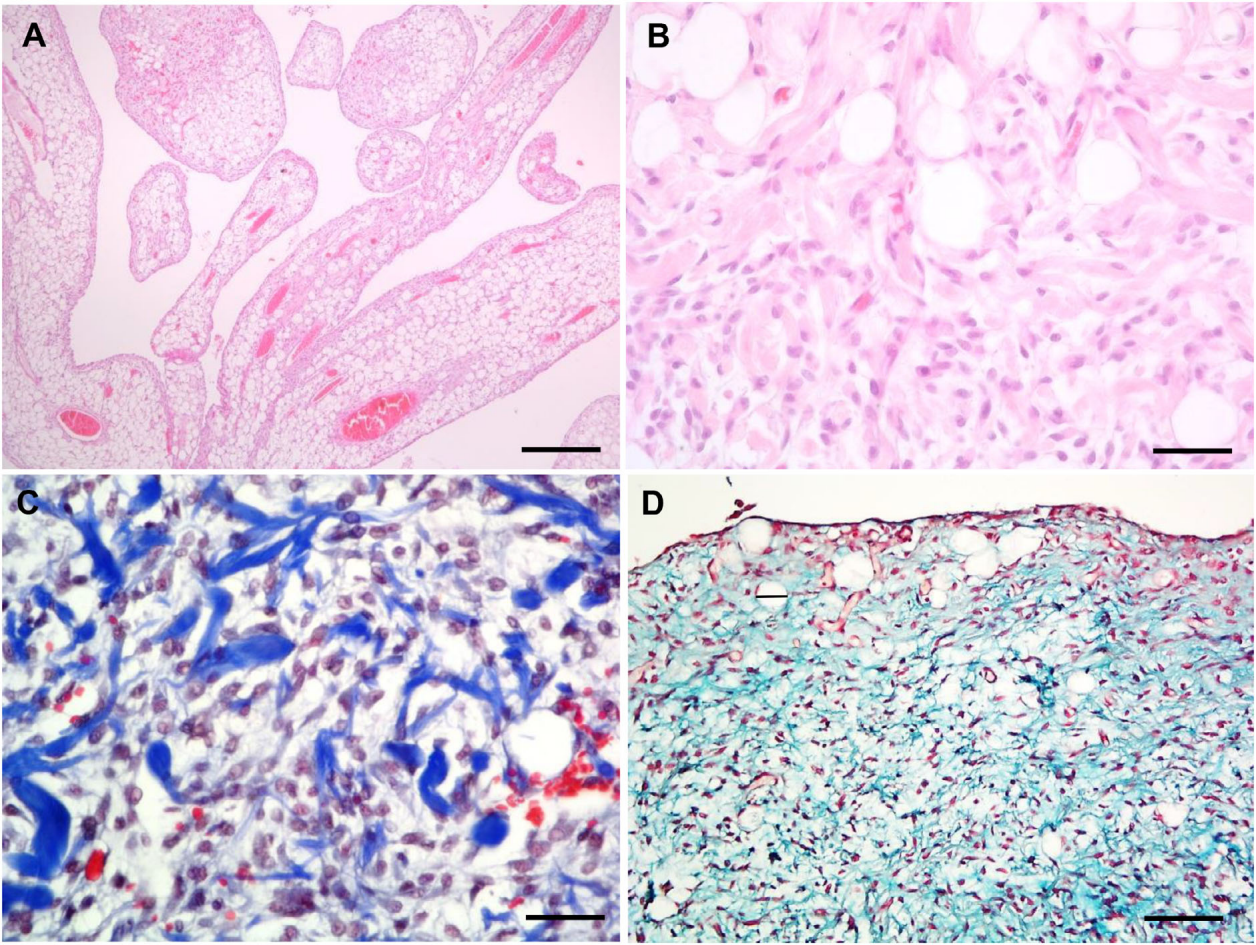
Histology of the canine spindle cell lipoma. (A) H&E, histological aspect of the papillae, with various blood vessels and surrounded by a palisading of spindle cells, bar = 600 µm. (B) H&E, concomitant presence of unilocular mature adipocytes and spindle cells, bar = 110 µm. (C) Masson trichrome, spindle‐cell population intermixed with ropey collagen fibres, bar = 110 µm. (D) Alcian blue, the myxoid matrix was highlighted by this stain, bar = 250 µm.

Histological examination revealed a partially demarcated, but not encapsulated, multilobulated neoplasm that extended to the deep surgical margins. The neoplasm was composed of well differentiated adipose tissue, occupying 70% of the tumour, arranged in lobules and multifocal forming papillary projections (Figure 2A); these were supported by a central core of delicate fibrovascular stroma and surrounded by a mixture of adipocytes and clusters of non‐cohesive spindle cells (Figure 2A). The stalks of papillae included medium‐sized blood vessels, occasionally containing blood or non‐organized fibrin thrombi. Mitotic figures were not observed in this cell population. The remaining 30% of the neoplasm was composed of parallel bundles, or less frequently haphazardly arranged spindle cells, mainly located at the periphery of the lobules and papillae and intermixed with numerous small‐sized blood vessels. These cells had 20–25 µm of maximum diameter, an intermediate to high nucleus/cytoplasmic ratio and a small to medium eosinophilic cytoplasm. The nuclei were pointed to oval, paracentral, 15 µm of diameter, with granular chromatin and occasionally a single and poorly evident nucleolus (Figure 2B). Mild anisocytosis and anisokaryosis were present. Four mitotic figures were observed in 10 HPF (in the areas with increased proliferative activity). Rare foetal‐like fat cells were identified intermixed with the spindle cells. Variable amounts of ropey collagen bundles and mucinous extracellular matrix were identified. The collagenous and myxoid nature of the matrix was confirmed by Masson trichrome and Alcian blue staining, respectively (Figure 2C,D). A moderate number of macrophages, containing intracytoplasmic brownish granular material (hemosiderin), associated with multifocal microhemorrhages and rare mast cells were also evident.

**FIGURE 3:**
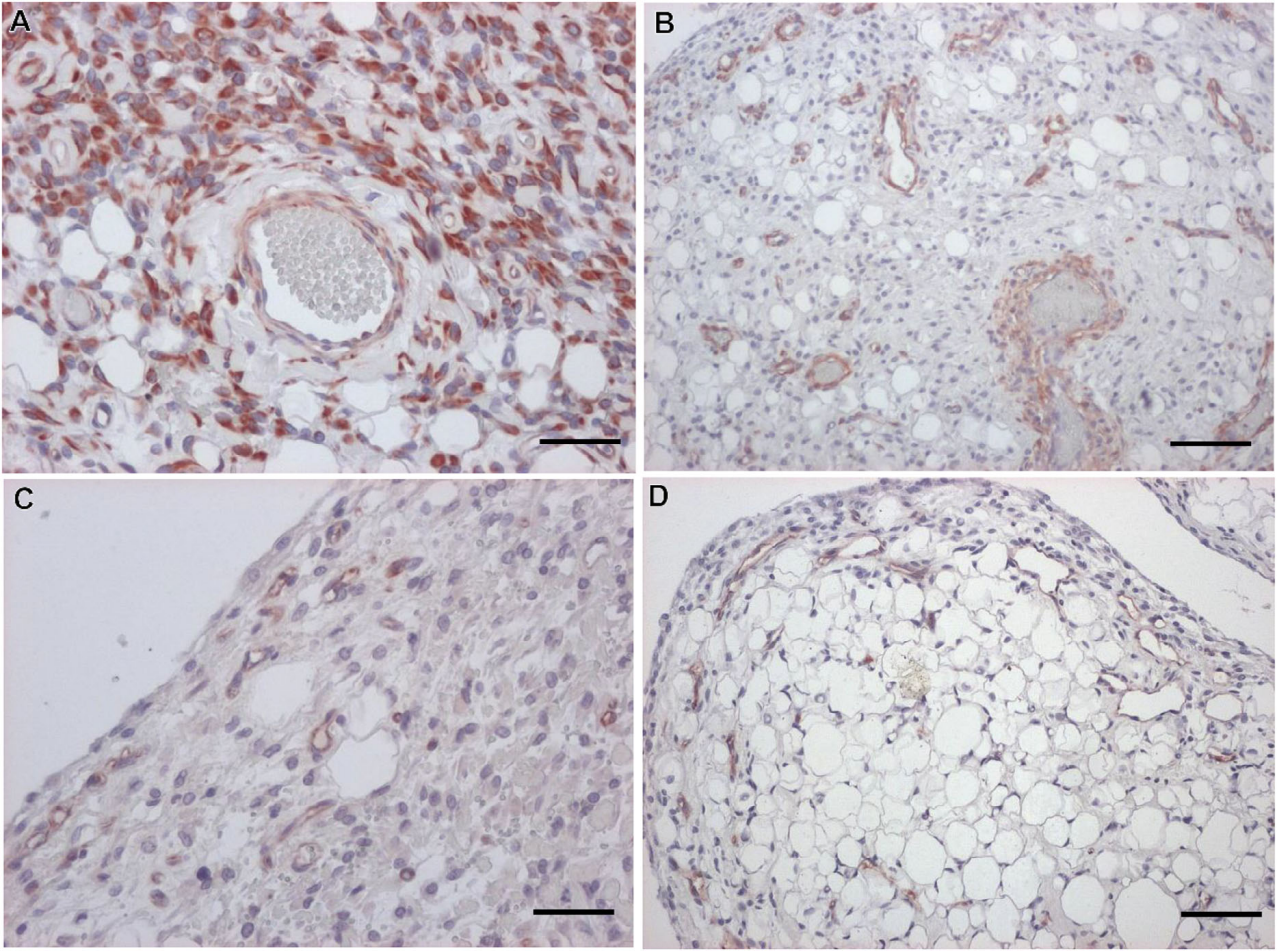
Immunohistochemistry of a canine spindle cell lipoma. (A) Intense and diffuse cytoplasmic expression of vimentin by perivascular neoplastic spindle cells and adipocytes, bar = 110 µm. (B) Spindle cells were negative to actin, contrasting with the muscular layer of vessels, which were positive, bar = 250 µm. (C) The spindle cells were negative to CD31, contrasting with the positivity of endotheliocytes in capillaries, bar = 250 µm. (D) No immunolabelling with the endothelial marker FVIII was observed in spindle cells, bar = 250 µm. Immunoperoxidase method, AEC chromogen, haematoxylin counterstain.

Immunohistochemistry was performed in order to better characterize the spindle cell population (Table 1). All neoplastic cells were intensely vimentin positive (Figure 3A). Alpha smooth muscle actin antibody stained the vascular wall (internal positive control) but not the spindle cells or adipocytes (Figure 3B). All spindle cells were negative for pancytokeratin, S‐100 and endothelial markers (FVIII and CD31—Figure 3C,D). These latter stained intra‐tumoural vessels (internal positive control) and highlighted the peripheral localization of small blood vessels in the papillae. It is opportune to mention that mature adipocytes were positive to vimentin but negative to alpha smooth muscle actin, FVIII and CD31. The histopathology diagnosis was spindle cell lipoma.

**TABLE 1:** Primary antibodies, specific dilutions, antigenic retrieval methods and results of the immunohistochemical study for spindle cells.

Antibody	Dilution	Antigenic retrieval	Spindle cells
Pan‐Actin^a^ (HHF35)	1:100	Water bath (100°C)	−
α‐Smooth Muscle Actin (1A4) ^a^	1:2000	Not treated	−
Vimentin^a^ (V9)	1:100	Not treated	+++
Desmin (DERII)^b^	1:400	Pepsin	−
NSE^a^	1:25	Not treated	−
S‐100 protein^a^	1:8000	Not treated	−
Pancytokeratin (AE1/AE3)^c^	1:50	Water bath (100°C)	−
CD31^a^	1:40	Pepsin	−
Factor VIII^a^	1:400	Pepsin	−

*Note*: For monoclonal antibodies, the specific clone is indicated between brackets. Score: (+++) = more than 70% of positive cells; (−) = negative.

^a^ Dako (Glostrup, Denmark).

^b^ Novocastra Laboratories (Newcastle, UK).

^c^ Zymed (San Francisco, CA).

The surgical wound healed by second intention, with granulation tissue. After 2 years, no signs of local relapse or distant metastasis were reported by the clinician.

## 3 Discussion

Herein, we presented a case of a slowly growing, large spindle cell lipoma in a young dog. This case presented mature adipose tissue and bundles of spindle cells producing thick collagen fibres and mucinous matrix, similar to previous reports (Gross et al. 2005; Avallone et al. 2017). However, compared to the published caseload (Avallone et al. 2017), the present case corresponded to a younger dog and presented a larger amount of mature adipose tissue. Additionally, the presence of papillae was distinctive histological finding of this case, comparing to those previously reported. Such papillary pattern has been described in humans, such as in lipoma arborescent or lipoma of the joint (Murphey et al. 2004), a proliferative, reactive lesion of the synovial connective tissue and in spindle cell lipoma, in which villiform projections appear in association with a severe mucinous neoplastic matrix (Fletcher et al. 2002). In the present case, we also observed a mucinous matrix that could partially justify the papillary formation. We further hypothesized that the growing of the tumour from the subcutis into spaces between the fasciae of gluteal muscles allowed the papillae formation since the presence of papillae in benign lipomatous tumours is associated with the presence of cavitary spaces. The single reference in the veterinary literature of papillae in benign lipomatous tumours corresponds to an angiolipoma of the pericardial sac of a young bull (Galofaro et al. 2005). Another new finding refers to the presence of multinucleated cells with mild atypia, which have not been previously described (Avallone et al. 2017). This may be accounted by the fact that these cells were rare, being best observed in cytology samples rather in histopathology (where they can be taken as mononucleated cells).

In this case, the greasy macroscopic appearance of the cytological smears suggested their lipomatous nature (Raskin et al. 2016). The microscopic examination of FNA evidenced a biphasic population composed mainly by mature adipocytes and fewer fusiform mesenchymal cells. Herein, the use of OR in cytological smears was useful because it confirmed the lipidic nature of the cytoplasmic vacuoles observed in both cell populations (Raskin et al. 2016). The major cytological features of this canine spindle cell population are comparable to those described in human spindle cell lipoma (Lin and Zakowski 2008; Åkerman and Domanski 2012; Qian 2014). The ropey collagen fibres are considered a major diagnostic hallmark of this type of lipoma (Åkerman and Domanski 2012). Interestingly, we also observed some nuclear pleomorphism in spindle cells, including multinucleated cells with the nuclei peripherally arranged. These cytological features are more characteristic of human pleomorphic lipomas, being the multinucleated cells named floret cells (Lin and Zakowski 2008). In human pathology, spindle cell lipomas are typically positive to CD34 (Lin and Zakowski 2008; Qian 2014), being unknown if this also stands in the veterinary field. (We were unable to assess this in our case). In human pathology, pleomorphic and spindle cell lipomas are related variants that present overlapping clinical, morphological and genetic characteristics (Lin and Zakowski 2008; Qian 2014). This case suggests that canine spindle cell and pleomorphic lipomas, as occurred in human tumours (Qian 2014), may represent a morphological continuum. The presence of blood vessels in cytological preparations and the perivascular arrangement of some spindle cells were noteworthy features of the present case. These findings confirmed a previous histological description of canine spindle cell lipoma (Gross et al. 2005). It should be stressed that the abundance of mature adipose tissue and the rare foetal‐like fat cells allowed us to exclude a myxoid liposarcoma (Hendrick 2017). Additionally, the follow‐up was unremarkable, with no recurrence or metastases documented, thus further confirming the benign nature of the tumour.

In conclusion, we described, for the first time, the cytological, histological and imunohistochemical features of a subcutaneous spindle cell lipoma with a characteristic papillary growth in a young dog. Despite cytology cannot be the single diagnostic tool for the primary classification of human and animal mesenchymal tumours, it plays an important role in triaging (Qian 2014). Animals with benign lesions are the ones that benefit the most from a cytological diagnosis. Alongside with sarcoma, we would recommend considering spindle cell lipoma when a cytological sample from a subcutaneous mass in a dog is characterized by a mixture of mature adipocytes and spindle cells in association with bundles of ropey collagen. To differentiate between these two, advanced diagnostic techniques should be used.

## Author Contributions

**Marta Santos**: conceptualization and investigation, original draft. **Ricardo Marcos**: data curation, review and editing. **João Oliveira**: data curation, review and editing. **Giancarlo Avallone**: data curation, review and editing.

## Ethics Statement

The authors confirm that the ethical policies of the journal, as noted on the journal's author guidelines page, have been adhered to and the appropriate ethical review committee approval has been received. The EU guidelines (Directive 2010/63/EU) for the Care and Use of Laboratory Animals were followed.

## Conflicts of Interest

The authors declare no conflicts of interest.

## Peer Review

The peer review history for this article is available at https://www.webofscience.com/api/gateway/wos/peer‐review/10.1002/vms3.70587.

## Data Availability

The data that support the findings of this study are available from the corresponding author upon reasonable request.
